# Clustering of lifestyle and health behaviours in Australian adolescents and associations with obesity, self-rated health and quality of life

**DOI:** 10.1186/s12889-023-15724-6

**Published:** 2023-05-10

**Authors:** Kabir Ahmad, Syed Afroz Keramat, Gail M. Ormsby, Enamul Kabir, Rasheda Khanam

**Affiliations:** 1 School of Business, Faculty of Business, Education, Law and Arts, University of Southern Queensland, Toowoomba, Australia; 2 Centre for Health Research, University of Southern Queensland, Toowoomba, Australia; 3 Present Address: School of Business, Faculty of Business, Education, Law and Arts, and Centre for Health Research, University of Southern Queensland, Toowoomba, Australia; 4 Faculty of Business, Education, Law and Arts, University of Southern Queensland, Toowoomba, Australia; 5 School of Mathematics, Physics and Computing, Faculty of Health, Engineering and Sciences, University of Southern Queensland, Toowoomba, Australia

**Keywords:** LSAC, Adolescents, Latent class analysis, Cluster analysis, Health-related behaviours, Obesity, Self-rated general health, Health-related quality of life

## Abstract

**Objective:**

The primary aim of this study was to identify clusters of lifestyle and health behaviours and explore their associations with health outcomes in a nationally representative sample of Australian adolescents.

**Methods:**

The study participants were 3127 adolescents aged 14–15 years who participated in the eighth wave of the birth cohort of the Longitudinal Study of Australian Children (LSAC). A latent class analysis (LCA) was performed to identify clusters based on the behaviours of physical activity, alcohol consumption, smoking, diet, eating disorders, sleep problems and weight consciousness. Multinomial logistic regression models were fitted to the following health outcome variables: obesity, self-rated general health and pediatric health-related quality of life, to investigate their associations with LCA clusters.

**Results:**

Based on the prevalence of health behaviour related characteristics, LCA identified gender based distinct clusters of adolescents with certain outward characteristics. There were five clusters for male and four clusters for female participants which are named as: healthy lifestyle, temperate, mixed lifestyle, multiple risk factors, and physically inactive (male only). Adolescents in the healthy lifestyle and temperate clusters reported low and moderately active health risk behaviours, for example, low physical activity, inadequate sleep and so on, while these behaviours were prevailing higher among adolescents of other clusters. Compared to adolescents of healthy lifestyle clusters, male members of physically inactive (OR = 3.87, 95% CI: 1.12 – 13.33) or mixed lifestyle (OR = 5.57, 95% CI: 3.15 – 9.84) clusters were over three to five times more likely to have obesity; while for female adolescents, members of only multiple risk factors clusters (OR = 3.61, 95% CI: 2.00 – 6.51) were over three time more likely to have obesity compared to their counterpart of healthy lifestyle clusters. Adolescents of physically inactive (b = -9.00 for male only), mixed lifestyle (b = -2.77 for male; b = -6.72 for female) or multiple risk factors clusters (b = -6.49 for male; b = -6.59 for female) had a stronger negative association with health-related quality of life scores compared to adolescents of healthy lifestyle clusters.

**Conclusion:**

The study offers novel insights into latent class classification through the utilisation of different lifestyles and health-related behaviours of adolescents to identify characteristics of vulnerable groups concerning obesity, general health status and quality of life. This classification strategy may help health policy makers to target vulnerable groups and develop appropriate interventions.

## Background

Overweight and obesity affect 25% of Australian children and adolescents, causing excess weight-related health and wellbeing problems and higher health care costs [1]. While many studies have linked children’s suboptimal health behaviours and lifestyle (for example, physical activity, smoking, alcohol consumption, diet and nutrition) to the development of chronic diseases [2–4], few have focused on patterns of health-related behaviours with respect to how risk behaviours cluster among the individuals and impact health [5–7]. For example, children who eat more and are physically inactive are more likely to become adults with obesity [8]. Adolescence is the transition stage from childhood to adulthood, as well as a critical developmental period, during which many health practices emerge or are discarded, which in turn influence subsequent behavioural and health trajectories [9, 10]. For instance, a large proportion of adolescents do not engage in the recommended levels of physical activity, while they lead a sedentary lifestyle for longer than recommended [11], get insufficient sleep, or engage in smoking or alcohol consumption [12]. Hence, exploring distinct clusters of health-related behaviours is vital to assess how these might affect adolescents’ long-term health [13].

Overweight and obesity in childhood are complex conditions [3, 14, 15]. Body mass index (BMI), as a stand-alone indicator, cannot adequately capture the nature of obesity and may not serve as a sufficient basis to develop appropriate interventions [14, 15]. In paediatrics, the Edmonton Obesity Staging System classifies the functional limitations of obesity by four domains: metabolic, mechanical and mental health and social milieu [16, 17]. Further, problem behaviour theory and health lifestyle theory suggest specific potential indicators (physical activity, sedentary behaviours, smoking, alcohol consumption, diet, eating disorders from stress, sleep and weight-control behaviours), which can be gauged through social surveys, to predict health conditions [18–20]. Both theories predict that a range of negative and risky behaviours would cluster to a set of choices if these behaviours result from an underlying tendency towards deviant behaviour [19]. If this is the case, many adolescents’ lifestyles and psychosocial behaviours are interrelated rather than having separate effects [12]. Hence, investigation should be carried out targeting the behaviours of physical activity, alcohol consumption, smoking, diet, eating disorders, sleep problems and weight consciousness, rather than a particular behaviour in isolation [9]. However, to the best of our knowledge, limited studies have explored the co-occurrence of these psychosocial and lifestyle factors as a whole.

From the perspective of comprehensive approach, as suggested by the World Health Organisation, it is necessary to consider how all possible patterns of risk behaviours, for example, patterns of eating, stress-related eating, trajectories of smoking or drinking and physical fitness risk behaviours, might affect an individual’s health [9, 21–23]. Latent Class Analysis (LCA) is an innovative statistical approach that utilises person-centred characteristics of categorical and cross-sectional indicators to identify distinct subpopulations. By examining varying response pattern assemblages, LCA yields unobserved (latent) classes of individuals to ascertain the most parsimonious and interpretable set of classes – representing groups of homogeneous individuals within the class, however, heterogeneous across different classes [24]. Recent literature shows that LCA has been used increasingly to identify latent subgroups of related characteristics of various morbidities including asthma and obesity [25, 26]. However, existing studies have mostly been conducted in European and North American settings and among adults, and available studies among children to identify patterns of health behaviours related to obesity have primarily used a subset of health behaviours [4, 25, 27–29].

In some countries, identifying specific clusters of the national population across age and gender groups has helped identify homogeneous groups that can be targeted for specific public health interventions or prevention strategies [4, 25, 27–29]. A cluster analysis study among Finnish adolescents (*n* = 6792) divided them into distinct subgroups based on health-related behaviors and psychosocial symptoms, and these subgroups tended to have persistent unhealthy lifestyle habits like low levels of physical activity, high BMI, and smoking [12]. Gender differences were also observed in two studies of adolescents and preschool children in the US and France, respectively, which were classified by gender, age, lifestyle and socioeconomic positions [30, 31]. A study conducted in the Netherlands (*n* = 4395) investigated the clusters of health-compromising and delinquent behaviours in adolescents and adults. The results revealed two relevant clusters (alcohol and delinquency) for young adolescents [32]. In most of these studies, relationships of the identified clusters with obesity, self-rated health and quality of life remain unclear [33]. Moreover, limited studies have been conducted in the Australian context [31].

Therefore, the primary aim of this study was to identify the clusters of health behaviours in a nationally representative sample of Australian adolescents and to explore the association of defined clusters of lifestyles and health behaviours with obesity, self-rated health and quality of life. Adolescents’ lifestyle characteristics and health behaviours include physical activity, diet, sedentary behaviour, smoking, alcohol consumption, sleep problems, eating disorders and concerns regarding weight gain. Given the previous findings of gender-related differences in the aforementioned variables [11, 12] and based on the findings that the model fit statistics are better suited for gender-based analyses, LCA models were analysed separately for male and female participants.

## Methods

### Study design and participants

This cross-sectional study utilised data of 3127 adolescents aged 14–15 years that was obtained from the eighth wave of the birth cohort of the Longitudinal Study of Australian Children (LSAC). The LSAC is a representative household survey of Australian children and adolescents, launched in 2004, that biennially collects information on the health (physical and socio-emotional) and learning development of Australian children from their birth based on the context of the bio-ecological framework of human development [20]. Adolescents who participated in this wave provided data on a variety of dimensions concerning lifestyles and health-related behaviours: physical activity, sedentary behaviours, alcohol consumption and smoking, sleep, eating disorders, concerns on weight gain, obesity status, self-rated general health and quality of life. A multistage sampling technique was used to select the LSAC respondents at wave 1 and then they were followed up at wave 8. Household was the primary sampling unit, and information was acquired from the children themselves. Details of the LSAC survey are available elsewhere [20].

### LCA variables

The LCA was performed using variables related to lifestyle and health-related behaviours of the adolescents. Based on the bio-ecological framework followed in the LSAC study, variables that are risk factors for obesity, self-rated health and quality of life [20] were selected. These variables were measured by the LSAC survey team using the LSAC questionnaire in the eighth wave survey, among which Branch Eating Disorder Test data were obtained using a validated questionnaire. All the responses of these variables were provided by the adolescents. Details of these variables are described ahead.

#### Physical activity

The LSAC collects data on the number of days the adolescents performed at least one hour of moderate-to-vigorous physical activity per week. From these data, we summed the total number of exercise hours per week. Existing literature recommends at least two hours of physical activity per week [33–35]. Based on this cut-off point, we categorised participants into the following groups: ‘less than two hours of physical activity per week’, ‘two to three hours of physical activity per week’ and ‘more than three hours of physical activity per week’.

#### Sedentary behaviour

Sedentary behaviour was measured based on two activities: the number of hours spent per week (including both weekdays and weekends) on electronic games (does not play, up to 3 h, more than three hours) and the frequency of sharing or posting content on social media (frequently or daily, weekly/monthly, never). The categories were defined based on the extent of hours spent on screen games or the level of engagement in social media. More frequent engagement in games or social media activity indicated higher sedentary behaviours.

#### Alcohol consumption

Data on alcohol consumption data were self-reported by the participants. The participants were classified into two categories based on their responses to alcohol consumption in the last four weeks: no (non-drinkers) and yes (drinkers).

#### Smoking

Participants were asked if they had smoked in the last four weeks; they were dichotomised as smokers or non-smokers based on whether they smoked cigarettes during the time period (yes = smoker; no = non-smokers).

#### Diet

Diet was assessed by the frequency of consumption of fruits and vegetables, skim/low/no-fat milk, high-fat food, full-cream milk products and high-sugar drinks. Following the observance of different dietary approaches, participants were categorised according to their dietary intake. Intake of fruits and vegetables and high-fat foods was categorised as ‘none’, ‘1–2 times a day’, ‘3–4 times a day’, ‘ ≥ 5 times a day’. Meanwhile, consumption of full-cream milk products, skim/low/no-fat milk and high-sugar drinks was categorised as ‘none’, ‘once a day’, ‘twice a day’, ‘thrice or more a day’. These categories were taken from the preferred classification of the LSAC based on the frequency of consumption per day.

#### Eating disorders

Stress-related eating or eating disorders are linked to an increased risk of obesity [23]. Hence, this study sought to consider these variables in the analysis. In wave 8 of the LSAC, the birth cohort children completed the Branched Eating Disorders Test questionnaire, which can identify partial syndrome eating disorders. The tool has high validity and reliability, which were originally validated in a community sample of adolescents and demonstrated high sensitivity and specificity for identifying eating disorder cases [36, 37]. This tool, meeting at least two of the three diagnostic criteria for anorexia nervosa or bulimia nervosa included in the Diagnostic and Statistical Manual – III R [37, 38], indicates the presence of a partial syndrome eating disorder. Based on this assessment, the LSAC survey determined whether the child has partial syndrome anorexia and/or bulimia. Further, to assess binge eating, the following question was asked in the LSAC survey: ‘How often did the child lose control of eating?’ Possible responses included ‘none’, ‘around once a week’ and ‘two or more days a week’.

#### Sleep

The LSAC measured sleep duration and sleep quality by asking the following questions: ‘On average, how much sleep do you get per night?’ and ‘During the last month, how well do you feel you have slept in general?’ Sleep quality was grouped as ‘very well’, ‘well’ and ‘not well’, and sleep duration was categorised as ‘less than 8 h’, ‘8–9 h’ and ‘greater than 9 h’.

#### Weight-control behaviours

Adolescents’ dieting behaviour and exercising to control weight were also measured in wave 8. The following questions were asked to the participants: how would you feel if you gained one or two kilos of weight (‘no concern’, ‘a little concerned’ and ‘would worry/upset me’). Participants were also asked about the frequency of having gone all day without eating to control weight (‘never’, ‘one day a week’ and ‘two or more days a week’) and the frequency of exercise to control weight (‘none’, ‘one to three days a week’ and ‘four or more days a week’).

### Health status-related outcome variables

Several health-related variables were measured in this study to compare the defined clusters of health status among adolescents. These variables are obesity, self-rated general health and health-related paediatric quality of life (PedsQL), among which PedsQL is a validated questionnaire used by the LSAC team [39]. All the responses of the variables, except the BMI measurements, were provided by the adolescents.

#### Obesity

Obesity was measured using the BMI score of the adolescents. Interviewers measured the respondent’s weight using Tanita body fat scales and height using laser stadiometer, as described in the data user manual [40]. These measurements were used to calculate the BMI. Then, the LSAC team categorised participants’ BMI scores based on the cut-offs suggested by Cole et al. (2000, 2007) for adolescents by age and sex as follows: underweight, normal weight, overweight and obesity. In this study, obesity was one of the key outcome variables.

#### Self-rated general health

Adolescents were asked to rate their general health on an ordinal scale as follows: ‘excellent’, ‘very good’, ‘good’, ‘fair’ and ‘poor’. These categories were then regrouped into two categories for this study as follows: excellent/very good as good health and good/fair/poor health as poor health for the regression analysis.

#### Health-related quality of life (HRQoL)

In the LSAC, adolescents’ physical, emotional, school and Social Functioning were measured using the validated questionnaire of Pediatric Quality of Life (PedsQL) inventory [39]. This study used these subscales of HRQoL as the outcome variable as it is a reliable and responsive measure of health outcomes of adolescents, details are described by Varni et al. [39]. The following subscales of the PedsQL were used in this study: (i) Physical Functioning, (ii) Emotional Functioning, (iii) School Functioning, (iv) Social Functioning and (v) Psychosocial Health Summary [41].

The Physical Functioning subscale assesses participants’ physical development. Parents were asked about how often their children experienced the following problems in the past month: a) difficulty walking more than one block, b) difficulty in running, c) difficulties in sport or exercise, d) difficulty lifting something heavy, e) difficulty taking a bath or showering by themselves, f) difficulty doing chores in the house, g) having aches or pains and h) having low energy levels. The Emotional Functioning subscale measures the frequency of negative emotional states such as sadness and anxiety displayed by the children. Parents were asked how often the study children experienced the following problems in the past month: a) feeling afraid or scared, b) feeling sad or blue, c) feeling angry, d) trouble sleeping and e) worrying about what will happen to them. The School Functioning subscale assesses school adjustment and performance of the children. Parents were asked how often the children experienced the following problems in the past month: a) difficulty paying attention in class, b) forgetting things, c) difficulty keeping up with school activities, d) missing school because of not feeling well, e) missing school to go to the doctor or hospital. The Social Functioning subscale measures children’s relationships with their peers. Parents were asked to rate how frequently children experienced the following problems in the past month: a) difficulty getting along with other children, b) other kids not wanting to be their friends, c) getting teased by other children, d) not being able to do things that other children their age can do, e) difficulty keeping up when playing with other children. The Psychosocial Health Summary subscale involved combining the scores on the Emotional Functioning and Social Functioning subscales.

To calculate the scale scores, children’s primary caregivers (in most cases, their mothers) were asked to rate each item on a five-point scale: Never (1), Almost never (2), Sometimes (3), Often (4), and Almost always (5). Items were reverse-scored and transformed to a 0–100 scale (1 = 100, 2 = 75, 3 = 50, 4 = 25, 5 = 0), where higher scores indicated a higher level of functioning. Average scores were then calculated to obtain scores on the Physical, Emotional, School and Social Functioning subscales and Psychosocial Health Summary subscale. Details of the questionnaire and the validity and reliability of the PedsQL inventory are described elsewhere [39, 41].

### Statistical analysis

Clusters of health-related behaviours were identified for 3127 adolescent male and female adolescents using LCA, a subcategory of structural equation modelling. This unsupervised machine learning algorithm is designed to handle large datasets and categorical variables, and it has features to determine the optimal number of clusters from a set of observed variables [42]. An advantage of using the LCA method in this study is that in contrast to the traditional approach of describing the variability of a single health behaviour, it provides a framework for describing heterogeneity among adolescents in terms of health behavioural indicators [24]. Thus, LCA was used to identify unobserved (latent) classes based on categorical indicators of lifestyles and health-related behaviours. This method designates each participant a ‘best’ class assignment based on their maximum likelihood of belonging to an identified distinct class. Participants within the same class are regarded as homogeneous based on the indicator variables [3, 43]. This distinction is a person-centred approach, as opposed to more traditional variable-centred approaches such as multiple logistic regression analysis [24]. Analyses were performed using the LCA procedure in STATA (version 16.0) software. Based on previous studies [24, 25], models with one to eight classes were tested to determine the optimal number of classes. No covariates were included in this procedure. To determine the optimal number of classes, Bayesian Information Criteria (BIC), Akaike Information Criteria (AIC) and the likelihood functions L^2^ (deviance statistics) values for each model were compared. The model with the lowest AIC, BIC and likelihood-ratio values and highest log-likelihood value was the best fit. LCAs found better model fits for identifying distinct clusters among male and female participants separately, rather than all adolescents. The study identified five significant clusters for male participants and four clusters for female participants, as shown in Table 1. The distributions of the item response probabilities were evaluated, and the identified classes were named based on characteristics that were more likely to reflect the members of the class. Participants were assigned to classes in which they had the highest probability of membership; that is, they exhibited the traits that are representative of that class.

**Table 1:** Model fit statistics for the LCA models

Model	N	Log likelihood	df	AIC	BIC	L^2^	% reduction in L^2^
***For male participants***							
One class	1606	-24527.86	38	49131.72	49336.22	25426.43	-
Two class	1606	-24041.07	74	48230.14	48628.37	24452.85	3.8
Three class	1606	-23755.00	98	47706.00	48233.39	23880.71	6.1
Four class	1606	-23568.01	138	47412.02	48154.67	23503.99	7.6
**Five class**^**a**^	**1606**	**-23403.70**	**175**	**47157.40**	**48099.16**	**23178.11**	**8.8**
Six class	1606	-23336.56	210	47093.12	48223.24	23043.83	9.4
Seven class	1606	-23225.17	249	46948.34	48288.33	22821.05	10.2
Eight class	1606	-23151.64	278	46859.28	48355.33	22673.98	10.8
***For female participants***							
One class	1521	-23868.59	38	47813.18	48015.61	25574.27	-
Two class	1521	-23472.68	75	47095.36	47494.89	24782.45	3.1
Three class	1521	-22745.39	115	45720.78	46333.4	23327.87	8.8
**Four class**^**a**^	**1521**	**-22475.41**	**133**	**45216.82**	**45925.33**	**22787.91**	**10.9**
Five class	1521	-22311.89	190	45003.77	46015.93	22460.87	12.2
Six class	1521	-22214.14	207	44842.28	45944.99	22265.37	12.9
Seven class	1521	-22135.76	244	44759.52	46059.33	22108.61	13.6
Eight class	1521	-22042.32	277	44638.64	46114.26	21921.73	14.3

^a^Based on the model fit characteristics, five class and four class LCA classifications were adopted for male and female participants, respectively

Abbreviations: *df *degrees of freedom, *AIC* Akaike Information Criterion, *BIC* Bayesian information criterion

Further, descriptive analyses of the responses for each of the 17 latent class variables of health-related behaviours were performed and presented by sex and cluster groups. The associations between the identified latent classes and BMI categories or general health status were evaluated using multinomial and binomial logistic regression adjusted for child age. Further, association between the identified latent classes and the HRQoL scores were also projected utilising linear regression models. The adjusted odds ratios (ORs) and 95% confidence intervals were reported. All analyses were performed stratified by sex. Data were analysed in STATA (version 16.0).

## Results

### Sample characteristics

Of the 3127 participants, 49% were female. Regarding health risk behaviours, approximately 2.37% of adolescents smoked cigarettes, whereas alcohol consumption was prevalent among 6.75% of adolescents. Male participants spent more time on exercise (> 3 h/week: 41.53%) and playing electronic games on weekdays (up to 3 h: 57.38%) and weekends (> 3 h/week: 58.78%). In contrast, female participants spent more time on social media on a daily (34.19%) or weekly/monthly basis (52.99%). However, consumption of fatty foods and high-sugar drinks was lower among girls. Meanwhile, girls were more likely to engage in weight control through exercises and skipping meals and to be more concerned about weight gain (see Table 2).

**Table 2:** Distribution of the attributes of the latent class variables by gender

		Total (*n* = 3127)			Male (*n* = 1606)		Female (*n* = 1521)	
Description	Value	n	%	n		%	n	%
Consumed alcohol in the last four weeks	No	2916	93.25	1500		93.4	1416	93.1
	Yes	211	6.75	106		6.6	105	6.9
Smoked in the last four weeks	No	3053	97.63	1581		98.44	1472	96.78
	Yes	74	2.37	25		1.56	49	3.22
Partial syndrome anorexia and/or bulimia	No	97.09	97.09	1568		97.63	1468	96.52
	Yes	91	2.91	38		2.37	53	3.48
Exercise hours per week	< 2 h/week	808	25.84	401		24.97	407	26.76
	2 h/week	1128	36.07	538		33.5	590	38.79
	> 3 h/week	1191	38.09	667		41.53	524	34.45
Weekday hours on e-games	Does not play on weekdays	1199	38.34	336		20.92	863	56.74
	Up to 3 h	1529	48.9	928		57.78	601	39.51
	More than 3 h	399	12.76	342		21.3	57	3.75
Weekend hours on e-games	Does not play on weekend days	883	28.24	127		7.91	756	49.7
	Up to 3 h	1128	36.07	535		33.31	593	38.99
	More than 3 h	1116	35.69	944		58.78	172	11.31
Frequency of sharing/posting on social media	Frequently or several times a day	1038	33.19	518		32.25	520	34.19
	On a weekly or monthly basis	1597	51.07	791		49.25	806	52.99
	Never	492	15.73	297		18.49	195	12.82
Sleep quality	Very well	868	27.76	478		29.76	390	25.64
	Well	1824	58.33	943		58.72	881	57.92
	Not well	435	13.91	185		11.52	250	16.44
Sleep duration	< 8 h	408	13.05	207		12.89	201	13.21
	8–9 h	1223	39.11	627		39.04	596	39.18
	> 9 h	1496	47.84	772		48.07	724	47.6
Frequency of fruit and vegetable consumption	None	336	10.75	201		12.52	135	8.88
	1–2 times/day	849	27.15	474		29.51	375	24.65
	3–4 times/day	1033	33.03	516		32.13	517	33.99
	≥ 5 times/day	909	29.07	415		25.84	494	32.48
Frequency of high-fat food consumption	None	608	19.44	287		17.87	321	21.1
	1–2 times/day	1599	51.14	774		48.19	825	54.24
	3–4 times/day	674	21.55	385		23.97	289	19
	≥ 5 times/day	246	7.87	160		9.96	86	5.65
SC had full-cream milk products	None	1082	34.6	491		30.57	591	38.86
	Once/day	1126	36.01	561		34.93	565	37.15
	Twice/day	618	19.76	344		21.42	274	18.01
	Thrice or more/day	301	9.63	210		13.08	91	5.98
SC had skim/low/no fat milk	None	1924	61.53	992		61.77	932	61.28
	Once/day	762	24.37	367		22.85	395	25.97
	Twice/day	301	9.63	157		9.78	144	9.47
	Thrice or more/day	140	4.48	90		5.6	50	3.29
Frequency of high-sugar drink consumption	None	1395	44.61	650		40.47	745	48.98
	Once/day	843	26.96	430		26.77	413	27.15
	Twice/day	498	15.93	284		17.68	214	14.07
	Thrice or more/day	391	12.5	242		15.07	149	9.8
Frequency of losing control of eating	None	2167	69.3	1264		78.7	903	59.37
	Around Once a week	598	19.12	264		16.44	334	21.96
	Two or more days a week	362	11.58	78		4.86	284	18.67
How would you feel if you gained one or two kilos of weight?	No concern	1542	49.31	1006		62.64	536	35.24
	A little concerned	806	25.78	391		24.35	415	27.28
	Would worry/upset me	779	24.91	209		13.01	570	37.48
Frequency of skipping meals throughout a day to control weight	None	2826	90.37	1503		93.59	1323	86.98
	One day a week	231	7.39	89		5.54	142	9.34
	Two or more days a week	70	2.24	14		0.87	56	3.68
Frequency of exercises to control weight	None	1678	53.66	949		59.09	729	47.93
	One to three days a week	922	29.49	399		24.84	523	34.39
	Four or more days a week	527	16.85	258		16.06	269	17.69

### Cluster profiles

**Fig. 1:**
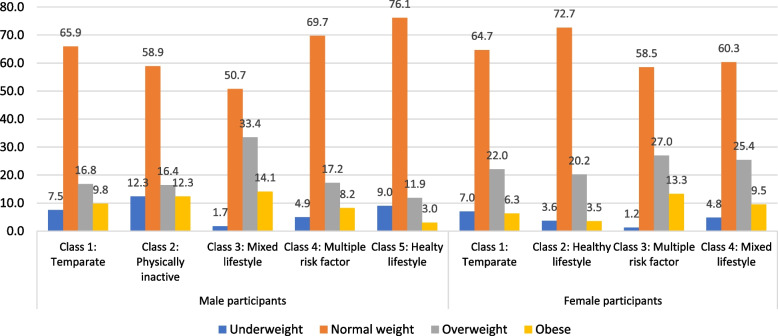
Percentage of BMI categories by clusters among male and female participants

**Fig. 2:**
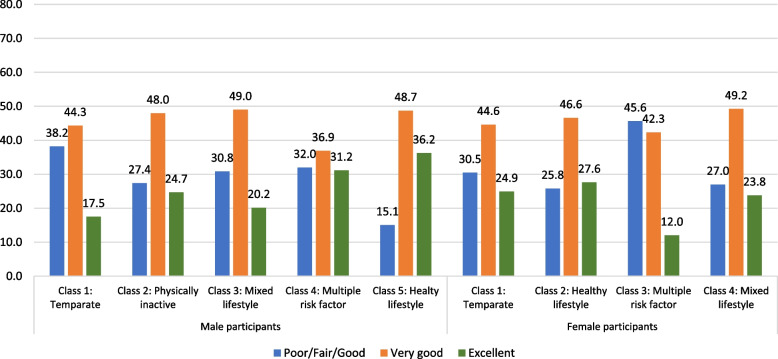
Percentage of general health categories by clusters among male and female participants

**Fig. 3:**
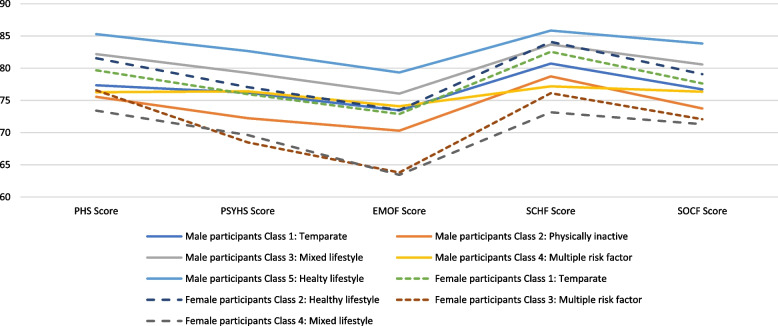
Pediatric quality of life average scores by clusters among male and female participants. Note: Abbreviations: PHS score, Physical Health Summary score; PSYHS Score, Psychosocial Health Summary score; EMOF Score, Emotional Functioning score; SCHF Score, School Functioning score; SOCF Score, Social Functioning score

The cluster analysis revealed a five-class model for male participants and a four-class model for female participants based on lowest BIC and lower AIC, likelihood-ratio (L^2^) and log-likelihood values compared to other models. The prevalence of 18 indicators across seven thematic areas, based on response probabilities of the defined clusters, is illustrated in Table 3. The clusters were named according to the indicators with high response probabilities as follows for male adolescents: i) temperate (27.4%), ii) physically inactive (4.6%), iii) mixed lifestyle (21.6%), iv) multiple risk factors (7.6%), and v) healthy lifestyle (38.9%); and for female adolescents: i) temperate (36.7%), ii) healthy lifestyle (43.3%, iii) multiple risk factors (15.8%) and iv) mixed lifestyle (4.2%). The healthy lifestyle cluster was the largest cluster for both boys and girls and was considered as the reference category while multinomial regression models were developed. Figure 1 shows that among different clusters, the proportion of adolescents with normal BMI was the highest (over 70%) among the healthy lifestyle clusters of both male and female participants. A higher number of adolescents in the mixed lifestyle and multiple risk factors clusters were with overweight (17–34%) or obesity (9–14%) compared to other clusters. Figure 2 shows the sex-based distribution of self-rated general health status across clusters, which reveals that poor health status (poor/fair/good) was less prevalent among adolescents in the healthy lifestyle cluster (26% or less) compared to adolescents in other clusters (27%–46%). Figure 3 shows the average scores on the five dimensions of the PedsQL among the clusters of male and female participants. Adolescents in the healthy lifestyle cluster obtained higher scores on all five dimensions compared to adolescents in other clusters.

**Table 3:** The prevalence of characteristics by lifestyle and health behaviours among the identified clusters for male and female participants

			**Clusters for male participants (*n* = 1606)**					**Clusters for female participants (*n* = 1521)**			
		Cluster #	**Cluster 1**	**Cluster 2**	**Cluster 3**	**Cluster 4**	**Cluster 5**	**Cluster 1**	**Cluster 2**	**Cluster 3**	**Cluster 4**
		Cluster Name	**Temperate**	**Physically inactive**	**Mixed lifestyle**	**Multiple risk factors**	**Healthy lifestyle**	**Temperate**	**Healthy lifestyle**	**Multiple risk factors**	**Mixed lifestyle**
		N (%)	**440 (27.4)**	**73 (4.6)**	**347 (21.6)**	**122(7.6)**	**624(38.9)**	**558 (36.7)**	**659 (43.3)**	**241 (15.8)**	**63 (4.2)**
**Behaviour group**	**Variable description**	**Categories**	%	%	%	%	%	%	%	%	%
Physical activity	Exercise hours per week	< 2 h	41.4	89.0	17.9	16.4	11.5	27.4	20.6	25.3	90.5
		2–3 h	33.6	4.1	43.8	33.6	31.1	41.9	38.1	42.3	4.8
		> 3 h	25.0	6.9	38.3	50.0	57.4	30.7	41.3	32.4	4.8
Sedentary behaviour	Weekday hours on e-games	Does not play on weekdays	0.2	20.6	25.7	17.2	33.7	5.9	99.4	55.6	65.1
		Up to 3 h	40.7	54.8	64.0	59.8	66.4	87.1	0.6	38.2	30.2
		More than 3 h	59.1	24.7	10.4	23.0	0.0	7.0	0.0	6.2	4.8
	Weekend hours on e-games	Does not play on weekend days	0.0	11.0	8.9	9.0	12.3	3.1	89.8	48.1	49.2
		Up to 3 h	3.9	35.6	36.0	27.9	53.4	75.1	10.2	34.9	36.5
		More than 3 h	96.1	53.4	55.0	63.1	34.3	21.9	0.0	17.0	14.3
	Frequency of sharing/posting on social media	Frequently or several times a day	33.0	2.7	36.9	45.1	30.1	29.4	34.3	53.9	0.0
		Weekly or monthly basis	47.3	4.1	52.7	45.1	54.8	58.4	58.0	40.3	1.6
		Never	19.8	93.2	10.4	9.8	15.1	12.2	7.7	5.8	98.4
Health risk behaviour	Alcohol consumption in the last 4 weeks	No	93.4	100.0	89.9	84.4	96.3	97.1	96.2	73.4	100.0
		Yes	6.6	0.0	10.1	15.6	3.7	2.9	3.8	26.6	0.0
	Smoking in the last 4 weeks	No	98.4	100.0	97.1	93.4	100.0	98.9	99.9	82.6	100.0
		Yes	1.6	0.0	2.9	6.6	0.0	1.1	0.2	17.4	0.0
Sleep	Sleep quality	Very well	28.4	9.6	20.5	30.3	38.1	31.9	27.8	10.8	4.8
		Well	57.5	24.7	69.5	57.4	57.9	60.6	62.2	52.7	9.5
		Not well	14.1	65.8	10.1	12.3	4.0	7.5	10.0	36.5	85.7
	Sleep duration	< 8 h	19.8	9.6	16.1	25.4	4.2	9.0	9.6	33.2	12.7
		8–9 h	42.1	43.8	43.5	32.0	35.3	39.1	40.5	37.3	33.3
		> 9 h	38.2	46.6	40.4	42.6	60.6	52.0	49.9	29.5	54.0
Diet	Frequency of fruit and vegetable consumption	None	16.8	100.0	3.8	10.7	4.5	4.1	2.7	12.9	100.0
		1–2 times/day	52.7	0.0	32.0	21.3	16.8	30.8	21.1	26.6	0.0
		3–4 times/day	24.3	0.0	43.2	19.7	37.7	35.0	35.5	36.5	0.0
		≥ 5 times/day	6.1	0.0	21.0	48.4	41.0	30.1	40.7	24.1	0.0
	Frequency of high-fat food consumption	None	12.7	98.6	13.8	0.8	17.6	10.9	21.7	22.4	100.0
		1–2 times/day	50.7	1.4	54.5	1.6	57.5	61.7	55.4	48.1	0.0
		3–4 times/day	30.2	0.0	28.2	16.4	21.5	20.4	19.3	19.9	0.0
		≥ 5 times/day	6.4	0.0	3.5	81.2	3.4	7.0	3.6	9.5	0.0
	SC had full-cream milk products	None	33.2	100.0	33.1	5.7	24.0	33.0	36.4	43.2	100.0
		Once/day	41.8	0.0	37.8	23.0	34.9	36.7	41.7	35.3	0.0
		Twice/day	13.6	0.0	23.9	30.3	24.2	23.3	16.8	13.7	0.0
		Thrice or more/day	8.4	0.0	5.2	41.0	16.8	7.0	5.0	7.9	0.0
	SC had skim/low/no fat milk	None	65.2	100.0	55.6	45.9	67.4	58.8	57.8	66.4	100.0
		Once/day	23.9	0.0	31.7	9.8	22.4	26.3	28.2	25.7	0.0
		Twice/day	6.4	0.0	12.7	20.5	9.6	11.3	11.2	2.9	0.0
		Thrice or more/day	4.6	0.0	0.0	23.8	6.6	3.6	2.7	5.0	0.0
	Frequency of high-sugar drink consumption	None	37.7	97.3	36.0	5.7	46.2	38.5	55.1	43.6	98.4
		Once/day	26.4	1.4	35.2	0.0	29.5	34.1	25.6	22.4	0.0
		Twice/day	26.1	1.4	18.7	14.8	13.8	16.7	11.5	18.3	1.6
		Thrice or more/day	9.8	1.4	10.1	79.5	10.6	10.8	7.7	15.8	0.0
Eating Disorder	Frequency of losing control of eating	None	87.3	97.3	40.1	80.3	91.7	66.7	66.8	11.6	100.0
		Around Once a week	10.2	2.7	44.7	13.1	7.4	23.1	20.6	28.6	0.0
		Two or more days a week	2.5	0.0	15.3	6.6	1.0	10.2	12.6	59.8	0.0
	Partial syndrome anorexia and/or bulimia	No	99.6	100.0	92.8	95.9	100.0	100.0	98.2	83.0	100.0
		Yes	0.5	0.0	7.2	4.1	1.0	0.0	1.8	17.0	0.0
Weight-control behaviours	How would you feel if you gained one or two kilos of weight?	No concern	68.0	50.7	17.3	80.3	82.1	43.9	38.1	5.0	44.4
		A little concerned	22.1	27.4	49.9	9.0	14.4	34.2	27.6	10.4	27.0
		Would worry/upset me	10.0	21.9	32.9	10.7	3.5	21.9	34.3	84.7	28.6
	Frequency of skipping meals throughout a day to control weight	None	94.8	98.6	85.6	90.2	97.3	94.3	95.3	44.4	98.4
		One day a week	5.0	1.4	11.8	6.6	2.7	5.7	3.8	35.3	0.0
		Two or more days a week	0.2	0.0	2.6	3.3	0.0	0.0	0.9	20.3	1.6
	Frequency of exercises to control weight	None	75.9	97.3	8.1	51.6	72.6	53.4	48.6	19.9	100.0
		One to three days a week	18.9	2.7	55.0	36.9	12.5	35.3	33.2	44.4	0.0
		Four or more days a week	5.2	0.0	36.9	11.5	14.9	11.3	18.2	35.7	0.0

Table 4 presents the associations between cluster membership and obesity or general health status. Table 5 presents the association between cluster membership and pediatric quality of life (PedsQL) outcome scores. Based on the descriptive statistics and the results shown in Tables 4 and 5, specific findings are presented ahead.

**Table 4:** Associations between cluster membership and obesity or general health status

	Multinomial Model: Obesity Status						Binomial Model: General Health Status*		
**Cluster name**	Overweight			Obesity			Poor health		
	OR	95% CI	p-value	OR	95% CI	p-value	OR	95% CI	p-value
***Models for male participants’ clusters***									
Class 1 Temperate	1.34	0.93—1.93	0.121	**2.37**	**1.32—4.23**	**0.004**	**2.99**	**2.21 – 4.05**	** < 0.001**
Class 2 Physically inactive	1.66	0.60 – 4.62	0.328	**3.87**	**1.12 – 13.33**	**0.032**	**2.97**	**1.29 – 6.83**	**0.010**
Class 3 Mixed lifestyle	**3.88**	**2.75 – 5.49**	** < 0.001**	**5.57**	**3.15 – 9.84**	** < 0.001**	**1.85**	**1.32 – 2.60**	**0.025**
Class 4 Multiple risk factors	1.37	0.79—2.39	0.257	1.93	0.84 – 4.42	0.118	**2.19**	**1.38 – 3.47**	**0.001**
Class 5 Healthy lifestyle (ref.)									
***Models for female participants’ clusters***									
Class 1 Temperate	1.18	0.89—1.58	0.234	**1.79**	**1.03—3.12**	**0.040**	1.17	0.90 – 1.52	0.229
Class 2 Healthy lifestyle (ref.)									
Class 3 Multiple risk factors	**1.48**	**1.03- 2.12**	**0.032**	**3.61**	**2.00 – 6.51**	** < 0.001**	**2.16**	**1.57 – 2.98**	** < 0.001**
Class 4 Mixed lifestyle	1.21	0.36- 4.14	0.750	1.68	0.27 – 10.59	0.578	2.18	0.63 – 7.56	0.219

^*^For the binomial model, the outcome of poor health was determined when the adolescents were with good/fair/poor health, considering the very good/excellent health as reference

**Table 5:** Associations between cluster membership and the pediatric quality of life (PedsQL) outcome scores

**Clusters**	PHS Score		PSYHS Score		EMOF Score		SCHF Score		SOCF Score	
	b	95% CI	b	95% CI	b	95% CI	b	95% CI	b	95% CI
***Models for male participants’ clusters***										
Class 1 Temperate	**-6.93**	**-9.46 to -4.39**	**-5.80**	**-7.65 to -3.95**	**-5.39**	**-7.41 to -3.37**	**-4.30**	**-6.53 to -2.07**	**-6.30**	**-8.19 to -4.41**
Class 2 Physically inactive	**-9.17**	**-14.33 to -4.01**	**-10.10**	**-13.87 to -6.33**	**-8.78**	**-12.91 to -4.65**	**-6.52**	**-11.07 to -1.97**	**-9.66**	**-13.51 to -5.81**
Class 3 Mixed lifestyle	-2.51	-5.20 to 0.19	**-2.99**	**-4.96 to -1.02**	**-2.99**	**-5.15 to -0.84**	-1.74	-4.12 to 0.64	**-2.77**	**-4.78 to -0.76**
Class 4 Multiple risk factors	**-7.76**	**-11.77 to -3.75**	**-5.47**	**-8.4 to -2.54**	**-4.65**	**-7.86 to -1.44**	**-7.49**	**-11.04 to -3.94**	**-6.49**	**-9.48 to -3.50**
Class 5 Healthy lifestyle (ref.)										
***Models for female participants’ clusters***										
Class 1 Temperate	-1.50	-3.81 to 0.82	-0.82	-2.63 to 0.99	-0.38	-2.40 to 1.64	-1.19	-3.30 to 0.93	-1.13	-2.90 to 0.64
**Class 2 Healthy lifestyle (ref.)**										
Class 3 Multiple risk factors	**-4.47**	**-7.50 to -1.43**	**-8.26**	**-10.63 to -5.89**	**-9.42**	**-12.07 to -6.76**	**-7.44**	**-10.21 to -4.66**	**-6.59**	**-8.91 to -4.27**
Class 4 Mixed lifestyle	**-6.67**	**-12.01 to -1.33**	**-6.73**	**-10.91 to -2.55**	**-9.56**	**-14.23 to -4.89**	**-9.74**	**-14.63 to -4.85**	**-6.72**	**-10.80 to -2.64**

*Abbreviations*: *PHS Score* Physical Health Summary score, *PSYHS Score* Psychosocial Health Summary score, *EMOF Score* Emotional Functioning score, *SCHF Score* School Functioning score, *SOCF Score* Social Functioning score

For male participants, healthy lifestyle, temperate, physically inactive, mixed lifestyle and multiple risk factors clusters were identified.

i) Male participants in the healthy lifestyle cluster (*n* = 624, 38.9%) reported the lowest levels of health risk behaviours (no smoking and almost no alcohol consumption), higher physical activity (> 3 h/week: 57.4%), low sedentary behaviour (33.7% adolescents did not play e-games and 66.4% played e-games less than 3 h in a week), high sleep duration (> 9 h: 60.6%), healthy diet practices (78.7% adolescents ate fruits and vegetables more than three times a day), almost no eating disorders (see Table 3).
ii) Male participants in the temperate cluster (*n* = 440, 27.4%) reported moderate levels of health risk behaviours (6.6% consumed alcohol and 1.6% smoked cigarettes), physical activity (< 2 h exercise/week: 41.4% adolescents), sleep (< 8 h sleep: 19.8% adolescents), diet (only 30.4% adolescents consumed vegetables/fruits three or more times per day), eating disorder (12.7% adolescents reported losing control of eating one or more days per week) and being conscious of weight gain (32.1% of adolescents reported being concerned about weight gain); however, adolescents in this cluster reported higher levels of sedentary behaviour during weekends (96.1% adolescents spend more than three hours on e-games). Compared to the healthy lifestyle cluster, male participants in this cluster were two times (OR = 2.37, 95% CI: 1.32 – 4.23) more likely to be with obesity. Further, male participants in this cluster were three times (OR = 2.99, 95% CI: 2.21 – 4.05) more likely to be in poor general health, compared to those of healthy lifestyle cluster (see Table 4). In the case of the paediatric quality of life, male participants in this cluster were more likely to obtain lower scores (b =—6.93 for the Physical Health Summary score, b = -5.80 for the Psychosocial Health Summary score and b = -6.30 for Social Functioning score) compared to the healthy lifestyle cluster (see Table 5).
iii) Male participants in the physically inactive cluster (*n* = 73, 4.6%) had the lowest level of physical activity (89% of adolescents engaging in < 2 h of exercise/week) and inadequate sleep quality (65.8% adolescents reported not getting good sleep). Adolescents in this cluster also engaged in less healthy dietary practices (almost none of the participants consumed fruits or vegetables any day) and were less conscious of weight gain (almost none of the participants engaged in restrained eating or exercises to control weight). Compared to the healthy lifestyle cluster, adolescents in this cluster were four times (OR = 3.87, 95% CI: 1.12 – 13.33) more likely to be with obesity. Moreover, adolescents in this cluster were three times (OR = 2.97, 95% CI: 1.29—6.83) more likely to be in poor general health (see Table 4). In the case of the paediatric quality of life, male participants in this cluster were more likely to have lower scores (b = -9.00 for Physical Health Summary score, b = -9.81 for Psychosocial Health Summary score and b = -9.66 for Social Functioning score) compared to those of healthy lifestyle cluster (see Table 5).
iv) Male participants in the mixed lifestyle (*n* = 347, 21.6%) cluster reported a miscellaneous routine regarding physical activity, health risk behaviour, sedentary behaviour, high-quality sleep, healthy diet and weight-gain consciousness. For example, though a majority of adolescents in this cluster engaged in 2 h (43.8%) or more (38.3%) of physical exercise per week, 10.1% of adolescents consumed alcohol and 2.9% smoked cigarettes, accounting for the second-highest prevalence among all the clusters. Furthermore, though around half of the adolescents slept 8–9 h and consumed fruits or vegetables 3–4 times per day, over 80% of adolescents consumed high-fat food at least once per day (up to 4 times/day). On the contrary, over 80% of adolescents were concerned about weight gain, and over half of the adolescents engaged in exercise one to three days per week to control weight. However, around 60% of adolescents lost control of eating at least once a week, and a few adolescents (7.2%) had partial syndrome anorexia and/or bulimia. Male participants in this cluster were more likely to be either overweight (OR = 3.88, 95% CI: 2.75 – 5.49) or obese (OR = 5.57, 95% CI: 3.15 – 9.84) and were more likely to have poor general health (OR = 1.85, 95% CI: 1.32 – 2.60) compared to those in the healthy lifestyle cluster (see Table 4). Further, members of this cluster more likely to obtain lower HRQoL scores (b = -2.77 for Social Functioning score) compared to those in the healthy lifestyle cluster (see Table 5).
v) Male participants in the multiple risk factors (*n* = 122, 7.6%) cluster had the highest percentage of smokers (6.6%) and alcohol drinkers (15.6%) compared to those in other clusters. Male participants in this cluster had high levels of sedentary behaviour: over 80% played e-games 3 h or more on weekdays, and over 90% played e-games 3 h or more on weekends (see Table 3). They also engaged in social media more frequently and were indifferent about weight gain. Members of this cluster were more likely to have poor general health (OR = 2.19, 95% CI: 1.38 – 3.47) and more likely to obtain lower HRQoL scores (b = -6.49 for Social Functioning score) compared to those in the healthy lifestyle cluster (see Table 4 and 5).

For female participants, healthy lifestyle, temperate, mixed lifestyle and multiple risk factors clusters were identified.

i) Female participants in the healthy lifestyle cluster (*n* = 659, 43.3%) reported the lowest levels of health risk behaviours (no smoking and almost no alcohol consumption), higher physical activity (around 80% of adolescents exercising 2 h or more per week), low levels of sedentary behaviour (almost no adolescents playing e-games in the weekdays, and 89.8% did not play e-games on weekends), long sleep duration (90% adolescents slept 8 h or more per night) with good quality sleep, healthy diet practices (over 97% adolescents ate fruits and vegetables regularly) and almost no eating disorders (see Table 3).
ii) Female participants in the temperate cluster (*n* = 558, 36.7%) had moderately active health behaviours, physical activity (around 67% of adolescents engaged in 2 h or more exercise per week), moderate hours of sleep (39% of adolescents sleep 8–9 h and 52% of adolescents slept more than 9 h), healthy diet (65% adolescents ate fruit and vegetables 3 times or more per day), less eating disorders, moderate consciousness of weight gain and low levels of sedentary behaviour on weekends. Female participants were more likely to be with obesity (OR = 1.79, 95% CI: 1.03 – 3.12) in this cluster compared to those in the healthy lifestyle cluster (see Table 4).
iii) Female participants in the mixed lifestyle (*n* = 63, 4.2%) reported no smoking or alcohol consumption but engaged in less physical activity (90.5% of adolescents with less than 2 h of physical activity/week). The majority of adolescents in this cluster never used social media, and although their sleep duration was good, their sleep quality was not good. Further, they had a lower intake of inappropriate diet and low eating disorders and weight-gain consciousness. There were no significant associations between membership of the participants to this cluster and obesity or poor general health (see Table 4). Regarding the paediatric quality of life, female participants in the mixed lifestyle cluster were more likely to obtain lower HRQoL scores (b = -6.12 for the Physical Health Summary score, b = -6.46 for Psychosocial Health Summary score and b = -6.72 for Social Functioning score) compared to those in the healthy lifestyle cluster (see Table 5).
iv) Female participants in the multiple risk factors (*n* = 241, 15.8%) cluster reported multiple risks in various indicators, including physical activity or exercise (42.3% engaged in more than 2 h of physical activity but less than 3 h/week). Adolescents in this cluster had high sedentary behaviour, engaged in social media several times a day and had the lowest sleep quality and reported more eating disorders. Adolescents in this cluster were around four times more likely to be with obesity (OR = 3.61, 95% CI: 2.00 – 6.51) compared to those in the healthy lifestyle cluster. Further, they were two times (OR = 2.16, 95% CI: 1.57—2.98) more likely to have poor general health (see Table 4). Regarding the paediatric quality of life, female participants in this cluster were more likely to obtain lower HRQoL scores (b = -4.15 for the Physical Health Summary score, b = -8.09 for Psychosocial Health Summary score and b = -6.59 for Social Functioning score) compared to those in the healthy lifestyle cluster (see Table 5).

## Discussion

This study revealed a distinct pattern of health behaviours among Australian adolescents and identified them in different clusters. These clusters were significantly associated with obesity, general health status and HRQoL. Both male and female participants in the healthy lifestyle clusters reported lower health risk behaviours and hence its members were less likely to have obesity, to have poor general health or to obtain lower HRQoL scores. On the contrary, the temperate cluster reported moderate levels of physical activity, sleep time, diet, eating disorder and weight-gain consciousness. However, adolescents in the unhealthy clusters (physically inactive, mixed lifestyle and multiple risk factors) reported the lowest levels of physical activity, high sedentary behaviour on weekdays, poor sleep quality, less healthy diet, low levels of consciousness regarding weight management and higher smoking and alcohol consumption rates. Clustering the distinct patterns of health-related behaviours is crucial since these behaviours affect both health and life expectancy [44].

This study identified specific clusters by gender concerning obesity, self-perceived general health status and pediatric HRQoL. Boys from the temperate, physically inactive and mixed lifestyle clusters and girls from the mixed lifestyle and multiple risk factors clusters were more likely to be with obesity than their counterparts in the healthy lifestyle clusters. Previous studies shown that unhealthy health behaviours are associated with higher BMI [21, 33, 45–47], but there were no cluster wise identification of health risks. However, some cross-sectional studies indicated an unexplained inverse relationship [48, 49] or no association [50] of higher BMI with unhealthy energy balance-related or nutritional behaviours. The possible reasons for this inconsistency may be the nature and quality of data, as well as any geographical, behavioural or methodological differences.

The present study found that adolescent boys in the temperate, physically inactive and mixed lifestyle clusters and girls in the mixed lifestyle and multiple risk factors clusters were less likely to report very good or excellent general health than their counterparts with a healthy lifestyle. However, there are limited studies to corroborate this finding. A study conducted in Ireland found higher odds of negative perceptions about health in the unhealthy behaviour cluster than in the healthy cluster [4]. Furthermore, earlier studies have shown that a healthy lifestyle ensures very good or excellent general health [51–53]. Adolescents with adverse health practices may have unhealthy cardiovascular profiles and low peak bone masses, consequently deteriorating their general health [34].

The present study further revealed that male participants in the temperate and physically inactive clusters and female participants in the mixed and multiple risk factors clusters reported lower quality of life than did their healthy lifestyle counterparts. Unhealthy clusters, including those who engaged in minimal physical activity, sedentary habits, restrained diet and had lower dietary awareness, reported a reduced quality of life (HRQoL). Similar to previous study findings, individuals with undesirable lifestyles had a higher likelihood of poor HRQoL [4, 54, 55]. Possible reasons for this include body pain, inadequate energy supply to the body and psychosocial or emotional breakdown [53, 55]. In addition, clusters of unhealthy habits are associated with depression, anxiety, violent behaviours, insufficient social support and unpleasant perceptions of society, all of which can lead to poor HRQoL [54].

The present study also demonstrated that clusters with moderately healthy behaviour had a significant impact on male participants but not on female participants. The temperate cluster—denoted by moderate health practices—also showed increased body fat, poor health and lower HRQoL among male participants; these findings are supported by a study in Ireland setting [4]. Male participants were more vulnerable than female participants. However, there are limited studies to explain the causal relationships in these sex differences, suggesting the need for further large-scale investigations to consider unhealthy-to-moderate stability of lifestyles patterns through sex-stratified analyses.

The above discussed study findings have substantial public health implications. Firstly, utilisation of LCA enabled this study to classify the adolescents from the complex characteristics of several domains of health-related behaviours. Secondly, the study findings would enable the health policy makers to focus on developing and implementing interventions based on the lifestyle characteristics among adolescents. For example, policy makers may take up a multifaceted intervention approach to target multiple unhealthy behaviours, assuming this would be more effective than targeting a single risk factor at a time. Future studies should explore the effects of different treatments on these obesity-related clusters. In addition, future studies should investigate the impact of biological and family factors on health-related behaviour patterns over an extended period using further longitudinal data. The main strength of the present study lies in its relatively large sample size, focusing on a range of health risk behaviours. This is one of the first studies to cluster a variety of health behaviours among adolescents and assess their association with three different health outcomes. Another strength of the study is that it has utilised the eighth wave data of the LSAC to capture the health outcomes of adolescents with different healthy and unhealthy behaviours. Further advantages of this study include the use of validated and well-accepted measures to assess the outcome variables. For example, weight and height data to calculate participants’ BMIs were collected by trained professionals. Moreover, the LCA is an advanced statistical approach that ensures diverse benefits for more precise estimations [3].

The present study has some limitations. First, this study provides an overview of adolescents’ health behaviours and their association with obesity, self-rated health and HRQoL using unbalanced longitudinal data. This precludes causal inferences between the identified clusters and health outcomes. Second, the records of adolescents’ health behaviour patterns and general health status were self-reported by them. It might be possible that they failed to recall past events, leading to bias or over and underreporting of the results [3, 33]. Third, the weight control behaviour related variables might have reverse causality, especially for being associated with obesity in this cross-sectional study. Finally, although LCA is a powerful statistical procedure, it has few methodical limitations. LCA assigns individuals to classes based on their probability of being in classes, however, proper class assignment is not guaranteed. Eventually, while we analyse data based on the classes, the results are dependent on the choice of classes (made subjectively based on BIC, AIC and log-likelihood values). If a different choice would be made, that would lead to slightly different results. Furthermore, the properties of the identified classes of this study are complex, and they are assigned names based on the judgement of most prominent properties. Hence readers should be careful of the “naming fallacy” and need to be cautious to understand them properly [56].

## Conclusions

The current study identified sex-based clusters of obesity-related health risk behaviours among Australian adolescents. All unhealthy clusters were associated with increased obesity and lower levels of self-rated general health; however, the magnitude of the risk of poor health outcomes varied by the risk characteristics of the clusters. Understanding various lifestyle clusters and health-related risk behaviours may be important for policy makers when developing obesity prevention interventions. Future studies should investigate the effects of various interventions on reducing these obesity-related clusters. Identifying the associations of these clusters with morbidity and lower quality of life scores is important to determine health behaviour patterns in national and international settings, which may help with obesity prevention and improving the quality of life.

## Data Availability

The data analysed during this study were collected from the Longitudinal Study of Australian Children, managed by the National Centre for Longitudinal Data. The authors cannot share the data publicly, as there are some restrictions on the use of the data. Moreover, the data application’s approval is subject to a signed confidentiality deed. However, the data that support findings of this study are available at the National Centre for Longitudinal Data (NCLD), Australia. Anyone interested in accessing this data should contact the NCLD authority through the following email: ncldresearch@dss.gov.au, or complete an online application available in the following URL: https://growingupinaustralia.gov.au/data-and-documentation/accessing-lsac-data. Please contact the corresponding author (email: kabir_ahmad2000@yahoo.com) for further information the study data need to be accessed.

## References

[1] Australian Institute of Health and Welfare (2020). Overweight and obesity among Australian children and adolescents. vol. Cat. no. PHE 274.

[2] Abdel-Qadir HM, Lee DS (2007). The contribution of familial and heritable risks in heart failure. Curr Opin Cardiol.

[3] Anderson LN, Sandhu R, Keown-Stoneman CDG, De Rubeis V, Borkhoff CM, Carsley S, Maguire JL, Birken CS (2020). Latent class analysis of obesity-related characteristics and associations with body mass index among young children. Obes Sci Pract.

[4] Conry MC, Morgan K, Curry P, McGee H, Harrington J, Ward M, Shelley E (2011). The clustering of health behaviours in Ireland and their relationship with mental health, selfrated health and quality of life. BMC Public Health.

[5] Obel C, Linnet KM, Henriksen TB, Rodriguez A, Jarvelin MR, Kotimaa A, Moilanen I, Ebeling H, Bilenberg N, Taanila A (2009). Smoking during pregnancy and hyperactivity-inattention in the offspring–comparing results from three Nordic cohorts. Int J Epidemiol.

[6] O'Reilly JR, Reynolds RM (2013). The risk of maternal obesity to the long-term health of the offspring. Clin Endocrinol (Oxf).

[7] Ou L, Chen J, Hillman K, Eastwood J (2010). The comparison of health status and health services utilisation between Indigenous and non-Indigenous infants in Australia. Aust N Z J Public Health.

[8] Park MH, Falconer C, Viner RM, Kinra S (2012). The impact of childhood obesity on morbidity and mortality in adulthood: a systematic review. Obes Rev.

[9] Daw J, Margolis R, Wright L (2017). Emerging adulthood, emergent health lifestyles: sociodemographic determinants of trajectories of smoking, binge drinking, obesity, and sedentary behavior. J Health Soc Behav.

[10] Harris KM, Gordon-Larsen P, Chantala K, Udry JR (2006). Longitudinal trends in race/ethnic disparities in leading health indicators from adolescence to young adulthood. Arch Pediatr Adolesc Med.

[11] Tammelin T, Ekelund U, Remes J, Nayha S (2007). Physical activity and sedentary behaviors among Finnish youth. Med Sci Sports Exerc.

[12] Heikkala E, Remes J, Paananen M, Taimela S, Auvinen J, Karppinen J (2014). Accumulation of lifestyle and psychosocial problems and persistence of adverse lifestyle over two-year follow-up among Finnish adolescents. BMC Public Health.

[13] Schulenberg JE, Bryant AL (2004). O'MALLEY PM: Taking hold of some kind of life: How developmental tasks relate to trajectories of well-being during the transition to adulthood. Dev Psychopathol.

[14] Freedman DS, Wang J, Maynard LM, Thornton JC, Mei Z, Pierson RN, Dietz WH, Horlick M (2005). Relation of BMI to fat and fat-free mass among children and adolescents. Int J Obes (Lond).

[15] Sharma AM, Kushner RF (2009). A proposed clinical staging system for obesity. Int J Obes (Lond).

[16] Hadjiyannakis S, Buchholz A, Chanoine J-P, Jetha MM, Gaboury L, Hamilton J, Birken C, Morrison KM, Legault L, Bridger T (2016). The Edmonton Obesity Staging System for Pediatrics: a proposed clinical staging system for paediatric obesity. Paediatr Child Health.

[17] Hadjiyannakis S, Ibrahim Q, Li J, Ball GDC, Buchholz A, Hamilton JK, Zenlea I, Ho J, Legault L, Laberge A-M (2019). Obesity class versus the Edmonton Obesity Staging System for Pediatrics to define health risk in childhood obesity: results from the CANPWR cross-sectional study. Lancet Child Adolesc Health.

[18] Jessor R: Problem-behavior theory. In: Risikoverhaltensweisen Jugendlicher. edn.: Springer; 2001: 61–78.

[19] Cockerham WC (2005). Health lifestyle theory and the convergence of agency and structure. J Health Soc Behav.

[20] Soloff C, Lawrence D, Johnstone R: Technical Paper no. 1: Sample design. In: Growing Up in Australia. Australian Institute of Family Studies; 2005.

[21] Huh J, Riggs NR, Spruijt-Metz D, Chou CP, Huang Z, Pentz M (2011). Identifying patterns of eating and physical activity in children: a latent class analysis of obesity risk. Obesity (Silver Spring).

[22] Tabacchi G, Faigenbaum A, Jemni M, Thomas E, Capranica L, Palma A, Breda J, Bianco A (2018). Profiles of physical fitness risk behaviours in school adolescents from the ASSO project: a latent class analysis. Int J Environ Res Public Health.

[23] Jääskeläinen A, Nevanperä N, Remes J, Rahkonen F, Järvelin M (2014). Stress-related eating, obesity and associated behavioural traits in adolescents: a prospective population-based cohort study. BMC Public Health.

[24] Lanza ST (2016). Latent class analysis for developmental research. Child Dev Perspect.

[25] Ahmad K, Kabir E, Ormsby GM, Khanam R (2021). Clustering of asthma and related comorbidities and their association with maternal health during pregnancy: evidence from an Australian birth cohort. BMC Public Health.

[26] Madigan CD, Daley AJ, Kabir E, Aveyard P, Brown W (2015). Cluster analysis of behavioural weight management strategies and associations with weight change in young women: a longitudinal analysis. Int J Obes (Lond).

[27] Schuit AJ, van Loon AJ, Tijhuis M, Ocke M (2002). Clustering of lifestyle risk factors in a general adult population. Prev Med.

[28] Berrigan D, Dodd K, Troiano RP, Krebs-Smith SM, Barbash RB (2003). Patterns of health behavior in U.S. adults. Prev Med.

[29] Poortinga W (2007). The prevalence and clustering of four major lifestyle risk factors in an English adult population. Prev Med.

[30] Pate RR, Heath GW, Dowda M, Trost SG (1996). Associations between physical activity and other health behaviors in a representative sample of US adolescents. Am J Public Health.

[31] Saldanha-Gomes C, Marbac M, Sedki M, Cornet M, Plancoulaine S, Charles MA, Lioret S, Dargent-Molina P (2020). Clusters of diet, physical activity, television exposure and sleep habits and their association with adiposity in preschool children: the EDEN mother-child cohort. Int J Behav Nutr Phys Act.

[32] van Nieuwenhuijzen M, Junger M, Velderman MK, Wiefferink KH, Paulussen TW, Hox J, Reijneveld SA (2009). Clustering of health-compromising behavior and delinquency in adolescents and adults in the Dutch population. Prev Med.

[33] Leech RM, McNaughton SA, Timperio A (2015). Clustering of diet, physical activity and sedentary behaviour among Australian children: cross-sectional and longitudinal associations with overweight and obesity. Int J Obes (Lond).

[34] Boreham C, Riddoch C (2001). The physical activity, fitness and health of children. J Sports Sci.

[35] Butcher K, Sallis JF, Mayer JA, Woodruff S (2008). Correlates of physical activity guideline compliance for adolescents in 100 U/S. Cities. J Adolesc Health.

[36] Hughes EK, Kerr JA, Patton GC, Sawyer SM, Wake M, Le Grange D, Azzopardi P (2019). Eating disorder symptoms across the weight spectrum in Australian adolescents. Int J Eat Disord.

[37] Selzer R, Hamill C, Bowes G, Patton G (1996). The branched eating disorders test: Validity in a nonclinical population. Int J Eat Disord.

[38] O'Connor M, Warren D, Daraganova G: Eating problems in mid-adolescence. In: Growing Up In Australia – The Longitudinal Study of Australian Children, Annual Statistical Report 2017. edn. Edited by Warren D, Daraganova G. Melbourne: Australian Institute of Family Studies; 2018.

[39] Varni JW, Burwinkle TM, Rapoff MA, Kamps JL, Olson N (2004). The PedsQL™ in pediatric asthma: reliability and validity of the pediatric quality of life inventory™ generic core scales and asthma module. J Beh Med.

[40] Mohal J, Lansangan C, Howell L, Renda J, Jessup K, Daraganova G: The Longitudinal Study of Australian Children – Data User Guide, Release 8.0, October 2020. In: Growing Up in Australia. Melbourne: Australian Institute of Family Studies; 2020

[41] Susan Clifford, Sarah Davies, Alanna Gillespie, Katherine Lange, Melissa Wake: Longitudinal Study of Australian Children's Child Health CheckPoint Data User Guide – December 2018. In. Melbourne: Murdoch Children's Research Institute; 2018.

[42] Schreiber JB (2017). Latent class analysis: an example for reporting results. Res Social Adm Pharm.

[43] Schreiber JB (2017). Latent class analysis: an example of reporting results. Res Social Adm Pharm.

[44] Khaw K-T, Wareham N, Bingham S, Welch A, Luben R, Day N (2008). Combined impact of health behaviours and mortality in men and women: the EPIC-Norfolk prospective population study. PLoS Med.

[45] Liu J, Kim J, Colabianchi N, Ortaglia A, Pate RR (2010). Co-varying patterns of physical activity and sedentary behaviors and their long-term maintenance among adolescents. J Phys Act Health.

[46] Magee CA, Caputi P, Iverson DC (2013). Patterns of health behaviours predict obesity in Australian children. J Paediatr Child Health.

[47] Gubbels JS, Kremers SP, Goldbohm RA, Stafleu A, Thijs C (2012). Energy balance-related behavioural patterns in 5-year-old children and the longitudinal association with weight status development in early childhood. Public Health Nutr.

[48] Sanchez A, Norman GJ, Sallis JF, Calfas KJ, Cella J, Patrick K (2007). Patterns and correlates of physical activity and nutrition behaviors in adolescents. Am J Prev Med.

[49] van der Sluis ME, Lien N, Twisk JW, Steenhuis IH, Bere E, Klepp KI, Wind M (2010). Longitudinal associations of energy balance-related behaviours and cross-sectional associations of clusters and body mass index in Norwegian adolescents. Public Health Nutr.

[50] Cameron AJ, Crawford DA, Salmon J, Campbell K, McNaughton SA, Mishra GD, Ball K (2011). Clustering of obesity-related risk behaviors in children and their mothers. Ann Epidemiol.

[51] Harrington J, Perry IJ, Lutomski J, Fitzgerald AP, Shiely F, McGee H, Barry MM, Van Lente E, Morgan K, Shelley E (2010). Living longer and feeling better: healthy lifestyle, self-rated health, obesity and depression in Ireland. Eur J Public Health.

[52] Akpinar A (2017). Urban green spaces for children: A cross-sectional study of associations with distance, physical activity, screen time, general health, and overweight. Urban Forestry & Urban Greening.

[53] Sanders TAB (2004). Diet and General Health: Dietary Counselling. Caries Research.

[54] Chen X, Sekine M, Hamanishi S, Wang H, Gaina A, Yamagami T, Kagamimori S (2005). Lifestyles and health-related quality of life in Japanese school children: a cross-sectional study. Prev Med.

[55] Wu XY, Han LH, Zhang JH, Luo S, Hu JW, Sun K (2017). The influence of physical activity, sedentary behavior on health-related quality of life among the general population of children and adolescents: a systematic review. PLoS ONE.

[56] Weller BE, Bowen NK, Faubert SJ (2020). Latent class analysis: a guide to best practice. J Black Psychol.

